# Nanofibrous Scaffolds Incorporating PDGF-BB Microspheres Induce Chemokine Expression and Tissue Neogenesis *In Vivo*

**DOI:** 10.1371/journal.pone.0001729

**Published:** 2008-03-05

**Authors:** Qiming Jin, Guobao Wei, Zhao Lin, James V. Sugai, Samuel E. Lynch, Peter X. Ma, William V. Giannobile

**Affiliations:** 1 Department of Periodontics and Oral Medicine, School of Dentistry, University of Michigan, Ann Arbor, Michigan, United States of America; 2 Department of Biomedical Engineering, College of Engineering, University of Michigan, Ann Arbor, Michigan, United States of America; 3 Biomimetic Therapeutics, Inc., Franklin, Tennessee, United States of America; 4 Department of Biologic and Material Sciences, School of Dentistry, University of Michigan, Ann Arbor, Michigan, United States of America; 5 Michigan Center for Oral Health Research, School of Dentistry, University of Michigan, Ann Arbor, Michigan, United States of America; Center for Genomic Regulation, Spain

## Abstract

Platelet-derived growth factor (PDGF) exerts multiple cellular effects that stimulate wound repair in multiple tissues. However, a major obstacle for its successful clinical application is the delivery system, which ultimately controls the *in vivo* release rate of PDGF. Polylactic-co-glycolic acid (PLGA) microspheres (MS) in nanofibrous scaffolds (NFS) have been shown to control the release of rhPDGF-BB *in vitro*. In order to investigate the effects of rhPDGF-BB release from MS in NFS on gene expression and enhancement of soft tissue engineering, rhPDGF-BB was incorporated into differing molecular weight (MW) polymeric MS. By controlling the MW of the MS over a range of 6.5 KDa–64 KDa, release rates of PDGF can be regulated over periods of weeks to months in vitro. The NFS-MS scaffolds were divided into multiple groups based on MS release characteristics and PDGF concentration ranging from 2.5–25.0 µg and evaluated in vivo in a soft tissue wound repair model in the dorsa of rats. At 3, 7, 14 and 21 days post-implantation, the scaffold implants were harvested followed by assessments of cell penetration, vasculogenesis and tissue neogenesis. Gene expression profiles using cDNA microarrays were performed on the PDGF-releasing NFS. The percentage of tissue invasion into MS-containing NFS at 7 days was higher in the PDGF groups when compared to controls. Blood vessel number in the HMW groups containing either 2.5 or 25 µg PDGF was increased above those of other groups at 7d (p<0.01). Results from cDNA array showed that PDGF strongly enhanced *in vivo* gene expression of the CXC chemokine family members such as CXCL1, CXCL2 and CXCL5. Thus, sustained release of rhPDGF-BB, controlled by slow-releasing MS associated with the NFS delivery system, enhanced cell migration and angiogenesis *in vivo*, and may be related to an induced expression of chemokine-related genes. This approach offers a technology to accurately control growth factor release to promote soft tissue engineering *in vivo.*

## Introduction

Growth factors are essential for cellular signaling for migration, proliferation, differentiation, and maturation.[1] However, the development of an effective delivery system that integrates appropriate scaffolds and growth factors for specific tissue repair and regeneration presents a clinical challenge. [2] Recently, nanofibrous scaffolds with controlled-release growth factors have shown significant potential for tissue engineering applications.[3]

Platelet derived growth factor (PDGF) is a multifunctional growth factor family, composed of A, B, C, and D polypeptide chains which can form homo- or heterodimeric molecules, [4] which can then bind to two structurally related, intrinsic tyrosine kinase receptors (PDGF-Rα and PDGF-Rβ) to exert its biological effects. [5] PDGF not only participates in embryonic development of organs such as kidney, heart, and vasculature, [6] but also plays a very important role in postnatal tissue repair, regeneration and disease development. [7]–[9]

PDGF possesses biological functions on cellular chemotaxis, mitogenesis, proliferation, extracellular matrix synthesis, anti-apoptosis and vascularization. [6], [10]–[14] As a potential therapeutic agent, PDGF has been widely studied both preclinically and clinically. It has been shown that when PDGF-BB is locally delivered by peptide nanofibers, it not only prevented cardiomyocyte death and maintained systolic function after myocardial infarction preclinically; it also decreased infarct size after ischemia/reperfusion. [15], [16] Furthermore, pre-administration of PDGF-AB decreases the extent of myocardial injury in an aged rat myocardial infarction model. [17] These results are in part because PDGF promotes collagen synthesis, recruits mural cells through neovessels and regulates maturation of the infarct vasculature. [18] PDGF is FDA-approved for the treatment of neurotrophic diabetic ulcers[19] and for promoting bone repair of periodontal osseous defects, [8], [20] indicating that PDGF has an important impact not only on soft tissues, but also on osseous tissues.

Poly(L-lactic acid) (PLLA) and poly(lactide-co-glycolide) (PLGA) are biodegradable polymers with great potential for use in delivery of polypeptides and proteins, because, in addition to their biocompatibility, the release rate of polypeptides can be controlled by adjusting the factor loading, polymer molecular weight, lactide/glycolide ratio in the copolymer, and formulation methods.[21], [22] Recently, our group has developed a novel PLLA (poly-L-lactic acid) porous scaffold with the characteristic of PLLA nanofibrous pore walls (instead of the solid pore walls), which increases the scaffold porosity to 98%, favoring diffusion of nutrients and oxygen.[23] In addition, based on the finding that polymers with high molecular weight (HMW) degrade more slowly than those with low molecular weight (LMW), the *in vivo* release of growth factor embedded in microspheres is controlled by molecular weight,[22] which is different from the traditional simple coating method in which the release rate of growth factors depends on physico-chemical interactions between the adsorbed growth factors and scaffold surfaces.

In order to study the feasibility of PLLA nanofibrous scaffold (NFS)/microsphere (MS) constructs for tissue engineering and their effects on PDGF biological functions on tissue neogenesis and vascularization, PLLA NFS containing rhPDGF-BB encapsulated in PLGA MS of different MW were implanted subcutaneously *in vivo*, and the implant-tissue constructs were harvested and analyzed by histology, histomorphometry, cDNAarray, and quantitative real-time PCR.

## Materials and Methods

### Polymeric microsphere (MS) preparation

Poly(lactide-co-glycolide) microspheres with diameter in the sub-micrometer to nanometer range were fabricated using an established double emulsion technique.[23] Briefly, 100 µl of a 0.3 mg/ml PDGF-BB solution (in 20 mM sodium acetate, pH = 6.3) was emulsified into 1 ml of 10% PLGA with MW 6.4 kD or 65 kD in dichloromethane (DCM) solution, using a probe sonicator at 15 W (Virsonic 100, Cardiner, NY) for 10 s over an ice bath to form the primary water-in-oil (w/o) emulsion. The w/o emulsion was mixed with 20 ml of 1% PVA aqueous solution under sonication to form a water-in-oil-in-water (w/o/w) double emulsion. The emulsion was then magnetically stirred at room temperature (RT) for at least 3 h to evaporate dichloromethane then centrifuged to collect solid microspheres. The resultant microspheres containing PDGF-BB were washed twice with distilled water, freeze-dried, and stored at −80°C until use.

### Fabrication of poly(L-lactic acid) (PLLA) nanofibrous scaffolds

PLLA macroporous nano-fibrous scaffolds were fabricated by a combination of the phase separation and sugar-leaching techniques. Briefly, 600 µl of 10% PLLA/THF solution was cast under mild vacuum into an assembled sugar template by bound sugar spheres approximately 250–425 µm in diameter. The polymer/sugar composite was phase separated overnight at −20°C and then immersed for 2 days in cyclohexane to exchange THF. Following lyophilization, the sugar spheres were leached out in distilled water and highly porous scaffolds were formed. After re-lyophilizing, the scaffolds were cut into circular disks with dimensions of 7.2 mm in diameter and 2 mm in thickness. The average weight of the porous scaffolds was 2.5–3.0 mg each.

### Incorporation of PLGA microspheres into PLLA

PLGA microspheres containing PDGF-BB were incorporated into PLLA nano-fibrous scaffolds using a post-seeding method. Briefly, dry PLGA MS were suspended in hexane at a concentration of 5 mg MS/ml. Eighty microliters of the suspension were seeded onto each scaffold and the scaffold was air-dried for 30 min to evaporate the solvent. This procedure was repeated until one scaffold contained 2.5 or 25 µg rhPDGF-BB (BioMimetic Therapeutics, Inc., Franklin, TN). This calculation was based on a 77% encapsulation efficiency of PDGF-BB in the microspheres, as previously described [23]. The scaffold was then subjected to a mixed solvent of hexane/THF (volume ratio of 90/10) to immobilize the microspheres on the scaffold which was then vacuum-dried for 3 d to remove the solvent. The controls were scaffolds seeded with microspheres without PDGF-BB.

### PDGF scaffold implantation *in vivo*

All the animal experimental procedures were approved by the University of Michigan Committee of Use and Care of Animals.

In order to investigate the effect of PDGF release rate on PDGF biological function *in vivo*, nine groups of 3 animals each were prepared to test in a rat wound healing model. [24] They were grouped as follows:

Empty nanofibrous scaffold
Nanofibrous scaffold+65 kD microspheres without PDGF-BB
Nanofibrous scaffold+6.4 kD microspheres without PDGF-BB
Nanofibrous scaffold+2.5 µg PDGF-BB (simple coating)
Nanofibrous scaffold+25 µg PDGF-BB (simple coating)
Nanofibrous scaffold+6.4 kD microspheres containing 2.5 µg PDGF-BB
Nanofibrous scaffold+6.4 kD microspheres containing 25 µg PDGF-BB
Nanofibrous scaffold+65 kD microspheres containing 2.5 µg PDGF-BB
Nanofibrous scaffold+65 kD microspheres containing 25 µg PDGF-BB

Under isoflurane anesthesia, mid-sagittal incisions were made on the dorsa of Sprague Dawley rats (200 g weight from Harlan, Indianapolis, IN). Each scaffold implant construct was inserted into a surgical pocket in triplicate for each assay (using 3 different rats). The incisions were stapled closed. Assays included histologic analysis (*n* = 3 animals/group) and cDNArray/real time PCR analysis (*n* = 3 animals/group). Four blocks were placed in each animal, and the implants were harvested at 3, 7, 14, and 21 days.

### Histology, histomorphometry and immunohistochemistry

The harvested pellets were fixed in 10% neutral buffered formalin, embedded in paraffin, and longitudinally cut into 4- or 5-µm thick cross sections. Chosen sections were stained with hematoxylin and eosin to evaluate the nature of tissue neogenesis. Images of these specimens were captured using a Nikon Eclipse 50i microscope (Nikon, Inc., Melville, NY) fitted with a Nikon Digital Sight DS U1 camera (Nikon, Inc., Melville, NY) for analysis using Image Pro Plus™ software (Media Cybernetics, Silver Spring, MD). The entire area of the specimen and the area of tissue penetrating into scaffolds were measured. The remaining slides were used to perform Factor VIII-related antigen/von Willebrand factor immunohistochemical staining with an anti-human Factor VIII-related antigen/von Willebrand factor rabbit polyclonal antibody (NeoMarkers, Fremont, CA) and a DakoCytomation EnVision^+^® System-HRP(AEC) kit (Dako North America, Carpinteria, CA). The area and number of positive-stained blood vessels within the scaffolds were measured.

### RNA extraction and purification

After removal of the tissue-scaffold implants, pellets were directly placed into liquid nitrogen, pulverized into fine particles, and transferred into 15 ml centrifuge tubes (Becton Dickinson Labware, Franklin Lakes, NJ). Total RNA extraction was performed using TRIzol reagent (Invitrogen, Carlsbad, CA) according to manufacturer's protocol. In brief, 2 ml TRIzol was added into each tube, and the tubes were placed at RT for 20 min. After centrifugation at 3000 rpm for 15 min, the supernatants were transferred into new tubes. Following protein denaturation by chloroform addition and centrifugation, RNA was precipitated by isopropanol, washed with 75% Alcohol and dried. The RNA was then solublized and cleaned using DNase I and the RNeasy Mini Kit (QIAGEN Inc, Valencia, CA) according to provided protocols.

### Affymetrix GeneChip Analysis

**Figure 1:**
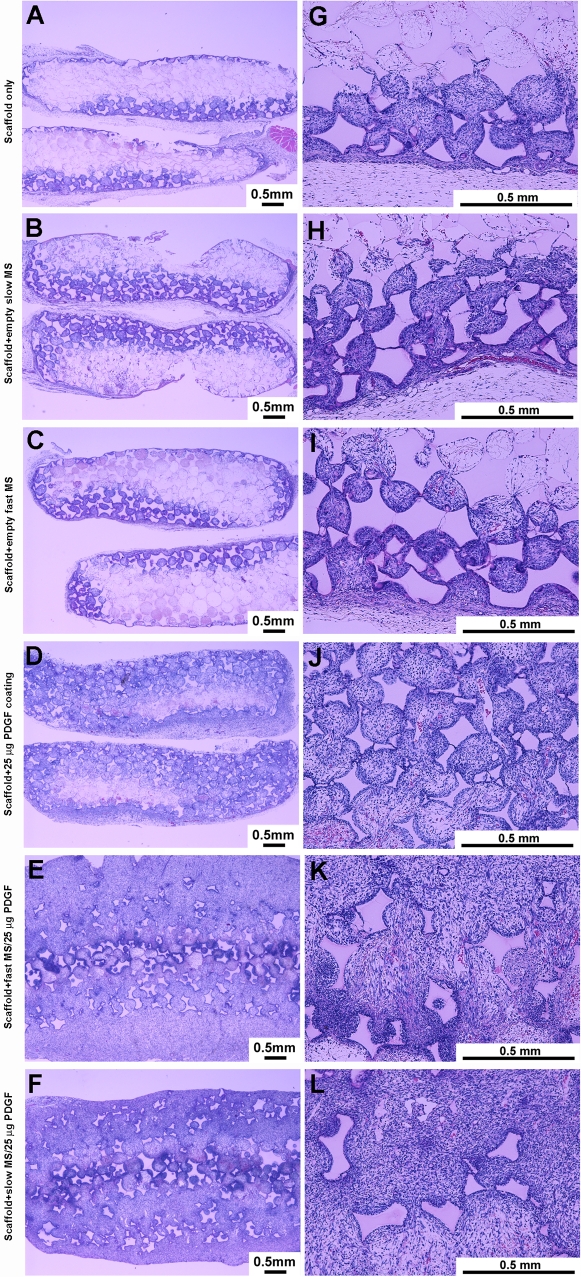
PDGF-containing microspheres (MS) in nanofibrous scaffolds (NFS) stimulates tissue invasion *in vivo.* Left panel at low magnification (2X) shows the tissue completely penetrated the entire NFS for the groups with PDGF encapsulated in PLGA microspheres, while other groups show a portion of scaffold occupied by ingrown tissue. In addition, the scaffolds were enlarged by the penetrated tissues in the groups with high dose PDGF encapsulated in PLGA microspheres. Right panel at high magnification (10X) demonstrated that most of the cells in the penetrated tissues were fibroblast-like cells and lymphocyte-like cells. On the scaffold pore surfaces multiple macrophage-like cells were seen. Bars indicate 0.5 mm.

**Figure 2:**
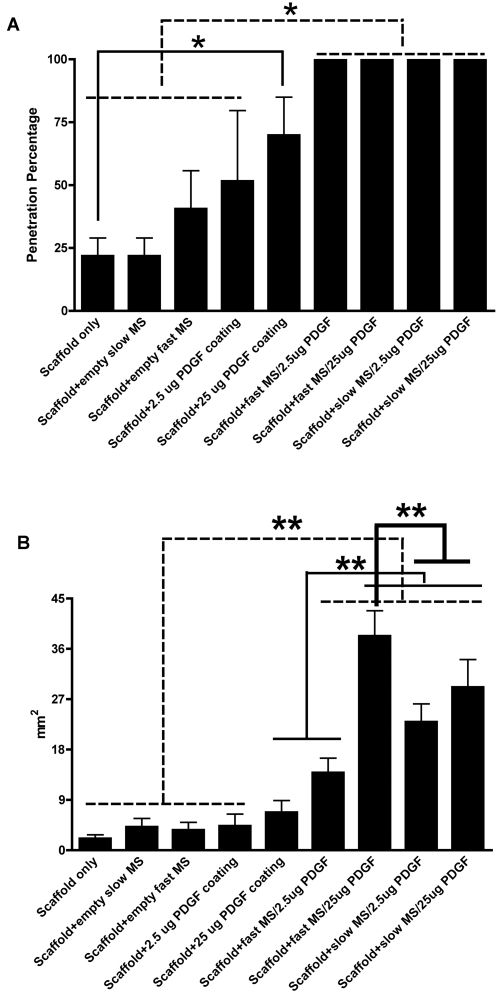
Histomorphometric analysis results of PDGF-inducing tissue penetration and neogenesis. *A* is percentage of tissue penetration. The percentage of tissue penetration within the groups of PDGF encapsulated in PLGA MS was statistically greater than that for the other groups with the exception of the 25 µg PDGF coating group. The percentage of tissue penetration in the 25 µg PDGF coating group is significantly greater than for the NFS only group. *B* demonstrates the areas for all groups. The areas in the groups for PDGF encapsulated in PLGA MS are statistically greater than those in the groups for NFS only, NFS containing empty, slow, and fast release PLGA microspheres, and 2.5 µg PDGF coating group. The groups with 2.5 µg PDGF encapsulated in slow release PLGA microspheres and 25 µg PDGF encapsulated in slow and fast release PLGA microspheres have larger areas than the groups with 25 µg PDGF coating and 2.5 µg PDGF encapsulated in fast release PLGA microspheres. The largest area was found in the group of 25 µg PDGF encapsulated in fast release PLGA microspheres. * indicates p<0.01, ** indicates p<0.05

From histology, the groups of NFS carrying 25 µg PDGF in MS had the most obvious tissue penetration and tissue neogenesis (Fig. 1 and 2). Thus, in order to screen the potential genes related to PDGF function *in vivo*, those two groups were selected, and 5 µg RNA from each of 3 specimens in each group was pooled together as the sample for Affymetrix GeneChip Analysis to screen for potential gene candidates to be subsequently assessed by quantitative real-time PCR. The group of empty NFS was used as a control.

Ten micrograms of total RNA was quantitatively amplified and biotin-labeled according to the Affymetrix GeneChip Expression Analysis Technical Manual. Briefly, RNA was converted to double-stranded complementary DNA (cDNA) using a SuperScript II RT kit (Invitrogen, Carlsbad, CA) with a T7-(T)_24_ primer (Proligo). The cDNA was then used for *in vitro* transcription in the presence of biotin-modified ribonucleotides (Enzo) to amplify single-stranded RNA. The biotin-labeled RNA was fragmented and 10 µg hybridized to a gene chip (Rat Genome 230 2.0 Array (Affymetrix, Santa Clara, CA)) at 45°C for 16 h. Chips were washed and stained with streptavidin R-phycoerythrin (Molecular Probes). After scanning the chips, the data were analyzed using Affymetrix GeneChip related software, Microarray Suite and Data Mining Tool.

### Quantitative Real-Time PCR

In order to verify the cDNArray results, quantitative Real-time PCR was performed using ABI Prism Sequence Detection System 7700 (Applied Biosystems, Foster City, CA). First, 1 µg total RNA was used as a template to generate cDNA with an oligo d(T) primer using the TaqMan Reverse Transcription Reagents kit (Applied Biosystems, Foster City, CA). Thermal conditions were: 25°C, 10 min; 48°C, 30 min and 95°C, 5 min. For the real time PCR, a 30 µl PCR reaction was prepared with 1 µl cDNA (RT product) and 1.5 µl mixture of gene specific probe (FAM dye) and primers from Applied Biosystems. The sequences for the *probes* are listed in Table 1. The PCR thermal conditions were: 50°C, 2 min; 95°C, 10 min followed by 40 cycles of 95°C, 15 sec and 60°C, 1 min. The ABI Prism Sequence Detection System 7700 and its operational software are capable of determining the linear phase of PCR reaction. An 18S primer and probe was used as an endogenous control.

**Table 1:** Real-time PCR probe sequences

Genes	Assay ID	Probe Sequence	Locus
CXCL1	Rn00578225_m1	TTGTCCAAAAGATGCTAAAGGGTGT	NM_030845
CXCL2	Rn00586403_m1	TCCAAAAGATACTGAACAAAGGCAA	NM_053647
CXCL5	Rn00573587_g1	GAGCTCAAGCTGCTCCTTTCTCGGC	NM_022214
CCL21b	Rn01764651_g1	GCTCCAAAGGCTGCAAGGGGACTGA	NM_001008513

### Statistical Analyses

The differences among groups for tissue penetration, tissue area, blood vessel number and gene expressions in real time PCR were statistically assessed by one-way analysis of variation (ANOVA) with Tukey multiple comparison post hoc test using a statistical software package Prism 4 (GraphPad Co. San Diego, CA). There was a minimum of three samples per group available for analysis. The level of significance was considered as p<0.05. For microarray assessments, triplicate samples were pooled for gene chip analyses. Samples were displayed using an arbitrary 10-fold change cut-off for subsequent assessment for quantitative real time PCR analysis.

## Results

### Histology and Histomorphometry

At 3 days post-implantation, no significant tissue ingrowth into the scaffolds was found in any of the groups (data not shown). However, by 1 week, penetrating tissue occupied the entire scaffold spaces in several groups containing PDGF encapsulated in MS, while the tissue penetration was seen only in the superficial regions of scaffolds in the groups with PDGF-coating only and the groups devoid of PDGF (Fig. 1). In addition, there was more vascularization and thicker connective tissue capsules surrounding scaffolds in the groups with PDGF encapsulation by microspheres than without PDGF containing MS. Not only did the NFS become larger in volume than their original shape, but also the porous structure of scaffolds appeared irregular and distorted, in contrast to the groups without PDGF encapsulated by MS. These findings were more noticeable in the groups of scaffolds with high dose PDGF MS than with low dose PDGF microspheres (Fig. 1). At 2 and 3 weeks, tissue invaded into the entire scaffold area for all groups (data not shown).

In parallel with the 1 week histological observations, the histomorphometry measurement results of 1 week specimens in Fig. 2 showed that the areas representing the specimen volume were significantly greater in the groups of NFS containing PDGF encapsulated in HMW MS and high dose PDGF encapsulated in LMW microspheres than the other groups. With regard to the tissue penetration percentage (penetration tissue area versus the whole area), the tissue invasion in the groups containing PDGF encapsulated by microspheres was greater than the groups with PDGF and the group with 2.5 µg PDGF coating.

### PDGF-BB MS in NFS Stimulates Neovascularization *in vivo*

**Figure 3:**
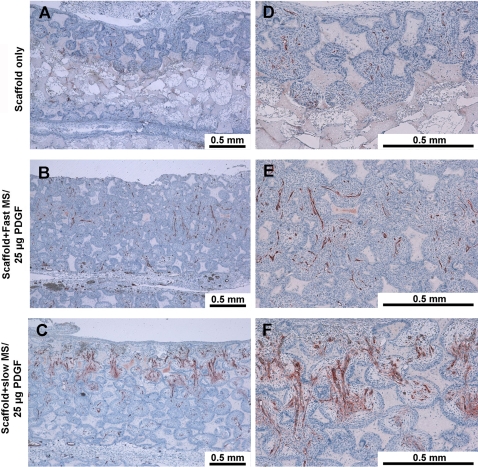
Nanofibrous scaffolds with PDGF microspheres promote vasculogenesis *in vivo.* Left panel is low magnification (10×), right panel is high magnification (40×). Positive Factor VIII stained blood vessels were located in the central regions of the pores within penetrated tissues. The blood vessels also permeated through the inter-openings between each pore. The group with 25 µg PDGF encapsulated in slow release PLGA microspheres had measurably more vascularization.

**Figure 4:**
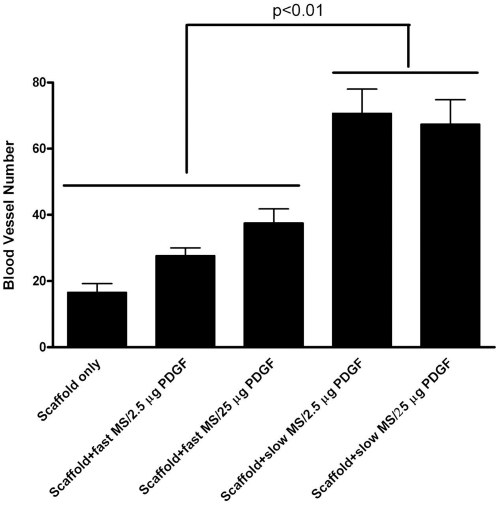
PDGF-containing microspheres in nanofibrous scaffolds increases angiogenesis *in vivo.* The blood vessel number was measured within each group using sections immunostained with Factor VIII antibody. Blood vessel number within both PDGF encapsulated, slow release groups was significantly greater that that in other groups. The blood vessel number of PDGF-encapsulated in the fast release PLGA group showed no difference from that in the NFS-only group. * indicates p<0.01

In order to explore the *in vivo* biological functions of PDGF delivered by microspheres in nanofibrous scaffolds on blood vessel formation, the vascularization within scaffolds was investigated using Factor VIII-related antigen/von Willebrand factor immunohistochemical staining. Figure 3 indicates that at 7d, vascularization forming inside scaffolds was greater in the groups of PDGF encapsulated in HMW microspheres, which is in accordance with the results of measurements of blood vessel number. There were significantly more blood vessels formed in the groups which contained NFS plus PDGF encapsulated in HMW microspheres (Fig 4). However, at 3, 14, and 21d, blood vessel number in the scaffolds displayed no significant difference among all groups (data not shown).

### cDNA array screening for potential PDGF-inducible genes

**Figure 5:**
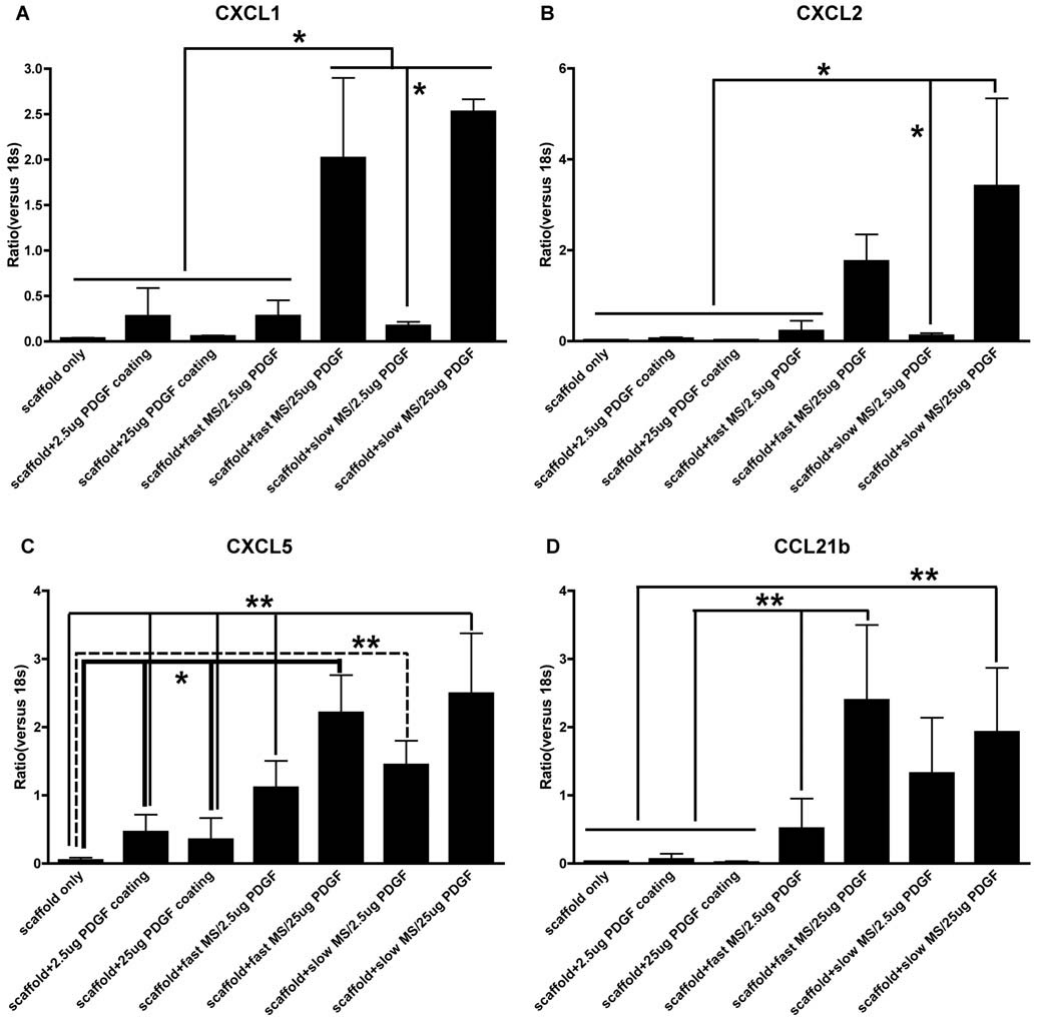
Chemokine gene induction in PDGF encapsulated microspheres *in vivo.* A: CXCL1 gene expression, B: CXCL2 gene expression, C: CXCL5 gene expression, D: CCL21b gene expression. * indicates p<0.01, ** indicates p<0.05.

In order to screen the potential gene expression changes induced by PDGF, RNA was extracted from 7 d specimens and used to perform cDNA array analysis. In order to target genes more sensitive to PDGF's effects, a 10-fold change in gene expression was used as the cut-off and results are displayed in Table 2. The cDNA array profiles demonstrate that PDGF primarily up-regulated the expression of three groups of potential genes. First, chemokine family genes such as CXCL1, 2, 5, and CCL 21b were up-regulated from 48.4-fold to 148.9-fold. However, CCL22 was down-regulated by 15.6-fold. Secondly, muscle-related or cell-backbone-related genes such as α-actin, myosin, and tropomyosin were increased 10.2- to 117-fold. Thirdly, interleukin-1 (IL-1) related genes such as IL-1alpha, beta, and IL-1 receptor were up-regulated by 12–15-fold. Cystatin E/M and carboxylesterase 3 were most prone to down-regulation by PDGF. The gene expression changes of chemokine and IL-1 family were confirmed by quantitative real-time PCR (Fig. 5, S1).

**Table 2:** Gene expression profile of PDGF-BB encapsulated microspheres in NFS vs. vehicle encapsulated microspheres in NFS. List of genes with greater than 10-fold change in expression from cDNArray analysis used as screening for subsequent qPCR analysis.

Gene	Fold Change
chemokine (C-X-C motif) ligand 1	148.95
actin, alpha 1, skeletal muscle	117.12
myosin, light polypeptide 2	86.93
similar to stefin A2 (predicted)	83.67
chemokine (C-X-C motif) ligand 5	66.65
chemokine (C-X-C motif) ligand 2	63.04
chemokine (C-C motif) ligand 21b (serine)	48.4
matrix metallopeptidase 3	47.66
Tryptophan hydroxylase 1	35.83
Transcribed locus	29.88
S100 calcium binding protein A9 (calgranulin B)	28.19
S100 calcium binding protein A8 (calgranulin A)	26.65
colony stimulating factor 3 (granulocyte)	26.22
gene model 1960, (NCBI)	25.67
similar to RIKEN cDNA 4933425K02 (predicted)	25.21
1388204_at	24.11
similar to MGC15476 protein (predicted)	23.44
sortilin-related receptor, L(DLR class) A repeats-containing (predicted)	21.17
Tropomyosin 1, alpha	21.12
EGF-like-domain, multiple 6	20.42
carbonic anhydrase 4	19.61
Transcribed locus, moderately similar to XP_574280.1 PREDICTED: similar to Ab2-143	19.08
Transcribed locus, moderately similar to XP_574280.1 PREDICTED: similar to Ab2-143	19.06
fast myosin alkali light chain	18.88
gene model 1960, (NCBI)	18.66
inhibin beta-A	17.59
interleukin 1 beta	15.24
neurotrophin receptor associated death domain	15.07
myoglobin	14.64
interleukin 1 receptor, type II	13.96
Transcribed locus	13.59
gene model 1960, (NCBI)	13.24
Transcribed locus	13.07
interleukin 1 alpha	12.83
Transcribed locus	11.94
ephrin A1	11.81
Transcribed locus	11.25
cAMP responsive element modulator	10.57
procollagen, type XI, alpha 1	10.57
1392736_at	10.55
breast cancer anti-estrogen resistance 1	10.38
galanin	10.2
myosin, heavy polypeptide 4, skeletal muscle	10.17
prokineticin 2	10.13
1385589_at	10.08
	Down-regulated
cystatin E/M	−50.08
carboxylesterase 3	−29.99
1375077_at	−20.03
elastase 1, pancreatic	−18.79
Similar to PIRB1 (predicted)	−17.71
CD5 antigen-like	−17.63
chemokine (C-C motif) ligand 22	−15.63
secretin receptor	−14.91
Similar to Ifi204 protein (predicted)	−10.96
thyroid hormone responsive protein	−10.77

Specimens harvested 7 days following treatment comparing the 25 µg PDGF-encapsulated in slow release PLGA microsphere group as compared to scaffold-only group are shown below.

### Quantitative Real-time PCR confirmation of PDGF-BB induction

Since most gene expression changes were found in chemokine family genes from the cDNArray profile, quantitative real-time PCR was used to investigate these genes in more detail. As shown in Fig. 5, CXCL 1 gene expression was higher in groups containing 25 µg PDGF encapsulated in high and low MW PLGA microspheres than in other groups, while higher CXCL2 gene expression was found only in the group containing 25 µg PDGF encapsulated in high MW PLGA microspheres, compared to all other groups. In addition, CXCL5 gene expression in the groups containing 25 µg PDGF encapsulated in PLGA microspheres was higher than that of the scaffold alone group and the two PDGF coating groups, while the group containing 25 µg PDGF encapsulated in HMW PLGA microspheres also had more CXCL5 gene expression, compared to 2.5 µg PDGF encapsulated in the LMW PLGA microspheres group. With regard to the CCL21b gene, 25 µg PDGF encapsulated in HMW PLGA microspheres group had stronger CCL21b transcription than the scaffold alone and both PDGF coating groups. The CCL21b gene expression within the group containing 25 µg PDGF encapsulated in LMW PLGA microspheres was higher compared to the scaffold alone group, the two PDGF coating groups, and the 2.5 µg PDGF encapsulated in LMW PLGA microspheres group. There was no statistically significant difference between HMW and LMW microspheres groups containing either 25 µg PDGF or 2.5 µg PDGF.

## Discussion

PDGF is a multiple biofunctional growth factor, which participates in cell migration, proliferation, chemotaxis, and vascularization. [6], [10], [14] The current study shows for the first time that a PLLA NFS/PLGA MS construct is favorable for PDGF delivery *in vivo* and promotes tissue neogenesis and vascularization. Furthermore, such *in vivo* PDGF functions have a close relationship with chemokine family members CXCL1, 2, 5, and CCL 21b and 22. Among them, CXCL1 has been shown to be a PDGF-induced early gene.[25]

Biological delivery systems for proteins and peptides can mainly be divided into two strategies: biomaterial delivery systems and gene delivery systems. For the biomaterial delivery system, there are two preparation methods: surface coating and encapsulation. In surface coating preparation, the release rate of proteins and peptides is primarily determined by the physico-chemical interactions between protein and biomaterial. In contrast, the release rate of proteins and peptides in encapsulation preparation occurs primarily via diffusion, polymer degradation, or a combination thereof [26]. It has been shown that release from low MW PLGA microspheres is diffusion controlled, whereas release from high MW PLGA microspheres typically occurs via a combination of diffusion and degradation. [27] Due to easier control for molecular weight than the surface interactions, delivery system by microspheres displays significant advantages. The gene delivery system is referred to the delivery by recombinant vectors such as recombinant plasmid, adenovirus, retrovirus and adeno-associated virus, which carry the genes encoding target proteins and peptides.

Our group has previously shown that *in vitro* release rate of PDGF encapsulated in PLGA microspheres can be controlled by PLGA MW. [23] HMW PLGA microspheres containing PDGF take a longer time to be degraded and consequently release PDGF more slowly, and vice versa. [23] In the present study, PLGA MW shows an obvious relationship to the *in vivo* effects of PDGF encapsulated by PLGA on tissue neogenesis and vascularization. The groups with PDGF encapsulated in PLGA microspheres had 100% tissue penetration, while the groups with PDGF coated on the surface of PLLA NFS resulted in only 20%–75% tissue penetration. Furthermore, specimen areas in the groups with 25 µg PDGF encapsulated in low and HMW PLGA microspheres and 2.5 µg PDGF encapsulated in HMW PLGA microspheres were much larger than other groups. These results indicate that tissue penetration and specimen area are dependent on *in vivo* PDGF release rate. In addition, the results of blood vessel number and area also showed a similar relationship with *in vivo* PDGF release.

We also explored the effects of delivery systems on *in vivo* PDGF function at the molecular level. The family of chemokines is composed of small molecular weight peptides with highly conserved cysteine motifs. Members of the chemokine family are categorized into four groups depending on the spacing of their first two cysteine residues. Their gene expression can be stimulated by many factors, including growth factors such as PDGF [25] and proinflammatory cytokines such as TNFα and IL-1.[28] These growth factor- and cytokine-initiated chemokine gene expression effects occur primarily through a phosphatidylinositol 3 kinase(PI3k)-Akt–Ikk-NF-κB pathway. [29], [30] After NF-κB is activated, NF-κB moves to the cell nucleus and controls the expression of numerous genes related to inflammation, tumor development, immune responses and tissue repair. [31], [32] Furthermore, Wood et. al. showed that the human CXCL1 gene has an NF-κB binding site (GGGAATTTCC) in its upstream promoter region, which is essential for IL-1 to induce CXCL1 promoter activity. [33] Therefore, the effects of PDGF on stimulating chemokine expression may depend on NF-κB.

A major role of chemokines is to guide the migration of cells but they also have roles in development, promoting angiogenesis and tracking cells to tissues that provide specific signals critical for cellular maturation. Certain inflammatory chemokines activate cells to initiate an immune response or promote wound healing [34]. CXC chemokines have two N-terminal cysteines, separated by one amino acid. CXC chemokines are subdivided into two groups, those with a specific amino acid motif of Glutamate-Leucine-Arginine (ELR) immediately before the first cysteine of the CXC motif (ELR-positive), and those without an ELR motif (ELR-negative). ELR-positive CXC chemokines specifically induce the migration of neutrophils, and interact with chemokine receptors CXCR1 and CXCR2. [35] These processes appear to be important in linking PDGF's effects during the inflammatory cascade associated with subsequent tissue neogenesis.

In our study, PDGF induced chemokine gene expression in both dose-dependent and duration-dependent manners. The CXCL1 gene expression induced by 25 µg PDGF encapsulated in low MW and high MW PLGA microspheres was higher than that in the scaffold alone group, PDGF coating group, and low dose PDGF microsphere group. In contrast, CXCL1 gene expression induced by 2.5 µg PDGF encapsulated in low MW and high MW PLGA microspheres had no significant differences from either the NFS alone or PDGF coating groups, which indicates up-regulation of CXCL1 gene expression stimulated by PDGF is dependent on PDGF dose. This relationship is also seen in chemokines CXCL2, CXCL5, and CCL21b. However, the gene expression of CXCL1, 2, 5, and CCL21b induced by 25 µg PDGF coated on PLLA scaffolds (not encapsulated in PLGA microspheres) was not different from that by the empty scaffolds or 2.5 µg (low dose) PDGF microsphere groups, which suggests that PDGF release from PDGF-coated scaffolds may be too rapid to be effective. Thus, the controlled delivery of PDGF will likely be critical for clinical success.[36]

Although PDGF induced expression of CXCL1, 2, and 5, the effects of these chemokines on PDGF function *in vivo* are still unclear. These chemokines seem not to influence PDGF functions on cell migration and proliferation because Nirodi et. al. reported [37] that there was no CXC receptor II (CXCR2) for CXCL1, 2 and 5 in normal human skin, while CXCR2 was found to be rich within endothelial cells, indicating these PDGF-inducible chemokines in the current study play a role in angiogenesis. In addition, CXCR2 is demonstrated to be the receptor responsible for ELR(+) CXC chemokine-mediated angiogenesis.[38] ELR motif: (glutamate-leucine-arginine motif) PDGF and CXC chemokines may have synergistic effects on angiogenesis, because the PDGF effects focus on pericytes and vascular smooth muscle cells and improve blood vessel growth, while ELR(+) CXC chemokine primarily affects endothelial cells. [38], [39]

In summary, a sustained release delivery system is key for *in vivo* PDGF application to promote tissue repair. *In vivo*, PDGF functions in a dose-dependent and release mode-dependent manner. A sustained release of PDGF not only influences tissue neogenesis and neovascularization, but also impacts the PDGF-induced gene profile of chemokine family members, actin, and interleukins. In addition, the chemokine family may be an important downstream factor for PDGF function. The use of controlled release NFS incorporating PDGF-BB encapsulated microspheres offers significant potential for soft and hard tissue engineering applications.

## Supporting Information

Figure S1 Interleukin 1 (IL-1) and CCL22 gene expressions induced with PDGF encapsulated microspheres in vivo. A: IL-1a gene expression, B: IL-1b gene expression, C: IL-1 receptor type II gene expression, D: CCL22 gene expression. * indicates p<0.01, ** indicates p<0.05.
(9.30 MB TIF)
