# Supplementary figures and images for: Nanofibrous Scaffolds Incorporating PDGF-BB Microspheres Induce Chemokine Expression and Tissue Neogenesis In Vivo
Source: PLoS One. 2008 Mar 5;3(3):e1729. doi: 10.1371/journal.pone.0001729 (PMC2248711; doi:10.1371/journal.pone.0001729)

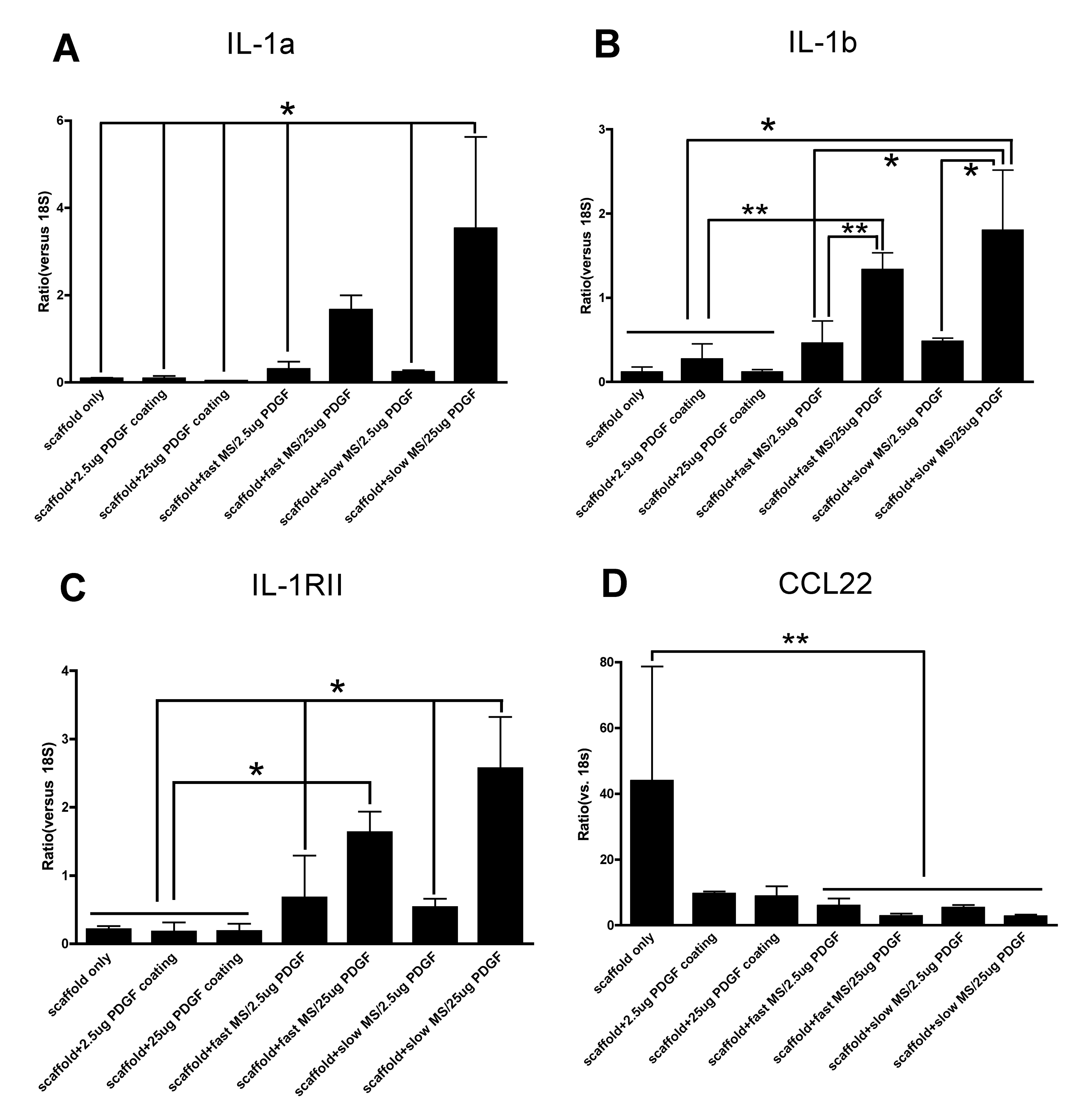

Supplement: Figure S1 — Interleukin 1 (IL-1) and CCL22 gene expressions induced with PDGF encapsulated microspheres in vivo. A: IL-1a gene expression, B: IL-1b gene expression, C: IL-1 receptor type II gene expression, D: CCL22 gene expression. * indicates p<0.01, ** indicates p<0.05. (9.30 MB TIF) [file pone.0001729.s001.tif]
